# Performance of immobilized denitrifying bacteria-enhanced ecological floating island for treating actual nitrogenous wastewater

**DOI:** 10.3389/fmicb.2026.1785618

**Published:** 2026-03-27

**Authors:** Juan Li, Yongqi Wang, Yuannan Long, Yan Liu, Yameng Zhang, Dian Zheng, Guanlong Yu

**Affiliations:** 1 School of Hydraulic and Ocean Engineering, Changsha University of Science and Technology, Changsha, China; 2 Hunan Polytechnic of Water Resources and Electric Power, Changsha, China; 3 Hunan Engineering Research Center of Intelligent Inspection and Digital Maintenance for Hydraulic Engineering, Changsha, China

**Keywords:** ecological floating island, immobilized denitrifying bacteria, microorganisms, nitrogen removal, plants

## Abstract

Excess nitrogen in water bodies can lead to eutrophication, posing a significant threat to aquatic ecosystems. Therefore, effective treatment of nitrogenous wastewater and the removal of nitrogen compounds from water bodies are essential for improving and maintaining water quality. In this study, the immobilized denitrifying bacterium *Alcaligenes faecalis* was employed to enhance the nitrogen removal efficiency of an ecological floating island (EFI). The research also aimed to determine the optimal operating conditions and assess the impact of different influent nitrogen concentrations on the treatment performance of the EFI. The results identified the optimal operating conditions for the integrated system as a C/N ratio of 16 and a DO concentration of 2 ~ 3 mg/L. Under this optimal condition, the system achieved a COD removal efficiency of 70.00 to 84.69% and a 100% total nitrogen (TN) removal efficiency across the entire tested range of influent TN concentrations (7.5 ~ 30 mg/L). Key microbial findings revealed that the highest microbial richness in the system was observed under the above optimal conditions; at the phylum level, the dominant microbial taxa were unclassified_k_norank_d_Bacteria and Proteobacteria, and at the order level, unclassified_p_Proteobacteria and unclassified_k_norank_d_Bacteria were the predominant groups, with unclassified_k_norank_d_Bacteria being the core functional taxa for nitrogen removal. Moreover, plant group 2 (*Canna indica L.* + *Thalia dealbata Fraser* + *Vallisneria natans*) and plant group 3 (*Canna indica L.* + *Myriophyllum verticillatum L.* + *Vallisneria natans*) exhibited significantly higher chlorophyll content and catalase activity than the other three plant groups, demonstrating that these two combinations are the optimal plant collocations with superior stress resistance and adaptability to the nitrogen-polluted wastewater environment. The study concluded that immobilizing denitrifying bacteria is an effective strategy for enhancing the water treatment performance of EFIs.

## 1 Introduction

With the advancement of global industrialization and urbanization, nitrogen pollution in water bodies has emerged as a pressing issue and a significant challenge to sustainable development. From cyanobacteria outbreaks in China’s Taihu Lake to the eutrophication of the Chesapeake Bay in the U. S. and the degradation of water quality in numerous rivers worldwide, nitrogen pollution has far-reaching consequences (Hu C. et al., 2025; Kang et al., 2024; Rattner et al., 2022). It disrupts aquatic ecosystems, threatens biodiversity, and accumulates through the food chain, thereby posing potential risks to human health (Javan et al., 2025). Traditional wastewater treatment methods, such as activated sludge, biofilm processes, and chemical precipitation, have helped mitigate wastewater pollution to some extent. However, these methods often suffer from limitations, including low treatment efficiency, high operational costs, significant sludge production, and the risk of secondary pollution (Raji et al., 2024). These methods are particularly inadequate for treating high-concentration nitrogenous wastewater, often failing to meet increasingly stringent environmental standards and treatment demands. As a result, the exploration of innovative wastewater treatment technologies, especially biological treatment methods, has become essential for addressing the nitrogen pollution challenge effectively.

The immobilized microbial system is formed by immobilizing microbial cells onto the carrier materials, effectively addressing issues such as microbial loss and low treatment efficiency (Hu Y. et al., 2025). Denitrifying bacteria, a group of microorganisms capable of reducing nitrate to nitrogen, play a central role in the wastewater denitrification process (Yang et al., 2023; Yu et al., 2024a). Immobilization technology involves immobilizing denitrifying bacterial cells onto carriers, preventing cell loss and increasing microbial concentration (Zhang Y. et al., 2024; Zhao et al., 2023). This approach enhances the stability and treatment efficiency of the system. Yu et al. (2019) used solid carbon sources and denitrifying bacterial spheres to remove organic matter and nitrogen from wastewater with low C/N ratios, achieving a ${\mathrm{NO}}_{3}^{-}-N$ removal efficiency of 96%. Similarly, Meng et al. (2024) employed biochar-immobilized aerobic denitrifying bacteria to treat low-nutrient wastewater, resulting in a nitrate removal rate approximately 20% higher than that of the control group. Furthermore, immobilized denitrifying bacteria exhibit excellent resistance to shock loads and environmental stress, maintaining high nitrogen removal activity even under extreme conditions (Li et al., 2022).

Ecological floating island (EFI) is an innovative treatment system that integrates aquatic plants and microorganisms to remove pollutants from water bodies through mechanisms such as plant uptake, microbial degradation, and physical adsorption (Arivukkarasu and Sathyanathan, 2023a). Aquatic plants provide surfaces for microorganisms to attach and grow, while enhancing the water’s microenvironment through oxygen transport via their root system, which promotes microbial growth and metabolism.

For conventional EFIs, microorganisms primarily attach to plant surfaces (e.g., roots and leaves) in an unconstrained manner, leading to low microbial concentration and poor retention—easily resulting in biomass loss and unstable denitrification efficiency (Arivukkarasu and Sathyanathan, 2023b). For standalone immobilized denitrifying bacteria bead systems: Their performance in actual wastewater with complex compositions and variable nitrogen concentrations has not been fully validated, restricting their industrial application. Nitrogen removal depends entirely on microbial denitrification, limiting the total nitrogen (TN) removal pathway and efficiency, especially in low-C/N wastewater (Yu et al., 2019). To address these inherent limitations of the two standalone technologies, this study innovatively integrates immobilized denitrifying bacteria *Alcaligenes faecalis* with EFI, and this strain is specifically selected for its prominent typical aerobic denitrification capacity that enables efficient nitrogen removal under aerobic conditions, a trait highly compatible with the micro-oxygen environment of the EFI system. The immobilization technology enhances microbial concentration and retention, overcoming the low biomass and instability of conventional EFIs. Meanwhile, aquatic plants in EFI provide synergistic effects (e.g., root oxygen secretion, nutrient uptake, and microenvironment regulation) to promote denitrifier activity, compensating for the single habitat defect of standalone immobilized bead systems. In this experiment, actual nitrogenous wastewater was selected as the treatment target. With the influent C/N ratio and DO levels adjusted to assess the treatment effectiveness of the immobilized denitrifying bacteria *Alcaligenes faecalis* in combination with the EFI. The study also aimed to investigate the device’s performance in treating wastewater with varying nitrogen concentrations. Meanwhile, we revealed the synergistic nitrogen removal mechanism of the coupled system, and identified its optimal plant combinations and core functional microbial taxa, from the perspectives of plant physiological and biochemical characteristics as well as microbial community structure and diversity. The findings aim to provide both theoretical and practical foundations for the engineering application of immobilized denitrifying bacteria combined with EFIs in the treatment of nitrogenous wastewater.

## 2 Materials and methods

### 2.1 Construction of ecological floating island

**Figure 1:**
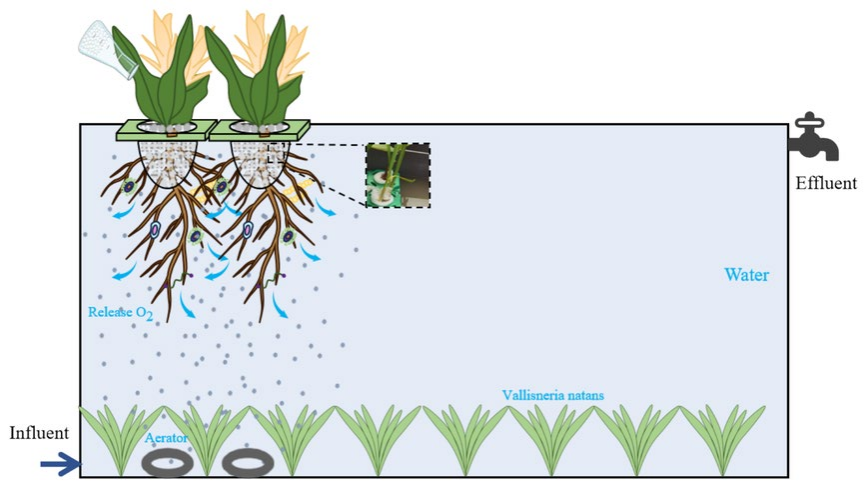
The experiments: schematic representation.

The EFI device was constructed from polyvinyl chloride, with dimensions of 3.1 m in length, 1.6 m in width, and 0.75 m in height, providing an effective water depth of 0.5 m and a total volume of 2.48 m^3^. An outlet valve was positioned 0.5 m from the bottom of the device. *Vallisneria natans* was planted at the bottom, while 80 small EFIs, each measuring 15 cm × 15 cm, were placed on the upper part of the device. The water inlet was located near the shore. A total of five plant groups were used in the experiment: Group 1 (*Canna indica L.* + *Pontederia cordata* + *Vallisneria natans*), Group 2 (*Canna indica L.* + *Thalia dealbata Fraser* + *Vallisneria natans*), Group 3 (*Canna indica L.* + *Myriophyllum verticillatum L.* + *Vallisneria natans*), Group 4 (*Thalia dealbata Fraser* + *Myriophyllum verticillatum L.* + *Vallisneria natans*), and Group 5 (*Iris pseudacorus L.* + *Pontederia cordata* + *Vallisneria natans*). Each EFI device integrates all five plant groups, with each group evenly distributed and collectively covering one-third of the water area. The plants were initially cultivated with Hoagland nutrient solution for 1 month, with water changes every 7 days. Once the plants reached a healthy growth stage, they were watered with raw water and transplanted into the EFI for the subsequent. The experiments. Schematic representation of the experimental setup as Figure 1.

### 2.2 Actual wastewater and experimental design

#### 2.2.1 Actual wastewater

In this study, the actual nitrogenous wastewater was collected from a small pond in the suburbs of Changsha, China. The suburban pond is a typical representative of natural eutrophic water bodies polluted by non-point source nitrogen, which is the main engineering application scenario of EFI technology for in-situ water remediation—with its inherent pollutant concentration ranges are provided in Table 1.

**Table 1:** Pollutant concentrations in actual water bodies.

Water quality indicators	${\mathrm{NH}}_{4}^{+}-N$	${\mathrm{NO}}_{3}^{-}-N$	TN	COD	TP
Concentration range (mg/L)	5	2.5 ~ 25	7.5 ~ 30	50 ~ 500	1.5

To meet the experimental design requirements for controlled parameters, the actual wastewater was not used directly but was moderately adjusted: Carbon source (glucose) was added to regulate the C/N ratio to the target values; KNO_3_ was supplemented to adjust the TN concentration to the preset setpoints while maintaining the ${\mathrm{NH}}_{4}^{+}-N$ concentration at 5 mg/L; No additional phosphorus was added.

#### 2.2.2 Experimental design

In this study, we investigated the effectiveness of immobilized denitrifying bacteria *Alcaligenes faecalis*, combined with an EFI, for treating actual nitrogenous wastewater. To this end, the immobilized *Alcaligenes faecalis* spheres were prepared as follows: 4 g polyvinyl alcohol and 2.5 g sodium alginate were mixed with 100 mL ultrapure water, heated to 85 °C with stirring until homogenized, sterilized (autoclave + ultraviolet), and degassed after cooling. A 2:1 volume ratio of bacterial suspension to carrier solution was blended, extruded via syringe into 2% (w/w) CaCl_2_ for 2 h crosslinking (≈5 mm beads), rinsed with sterile distilled water, and aged at 4 °C for 24 h. The immobilized spheres were subsequently dosed into the EFI system at a fixed 1% (w/v) mass fraction. This ratio ensures the microbial density required for efficient nitrogen removal in the system while avoiding hydraulic clogging and impaired mass transfer caused by the excessive addition of immobilized spheres.

To assess the system’s performance under different operating scenarios, the influent C/N ratio and DO levels were adjusted, and the device’s treatment efficacy for nitrogenous wastewater with varying concentrations was evaluated. For clarity, Table 2 explicitly delineates the correspondence between each EFI device (D1-D11) and its respective operating conditions.

**Table 2:** Experimental design parameters for EFI devices.

Device ID	Plant group	C/N	DO (mg/L)	Influent Concentrations (mg/L)			TP (mg/L)	HRT (d)
				TN	${\mathrm{NH}}_{4}^{+}-N$	${\mathrm{NO}}_{3}^{-}-N$		
D1	Group1 ~ 5	3	2 ~ 3	20	5	15	1.5	7
D2	Group1 ~ 5	7	2 ~ 3	20	5	15	1.5	7
D3	Group1 ~ 5	11	2 ~ 3	20	5	15	1.5	7
D4	Group1 ~ 5	14	2 ~ 3	20	5	15	1.5	7
D5	Group1 ~ 5	16	2 ~ 3	20	5	15	1.5	7
D6	Group1 ~ 5	16	3 ~ 4	20	5	15	1.5	7
D7	Group1 ~ 5	16	4 ~ 5	20	5	15	1.5	7
D8	Group1 ~ 5	16	5 ~ 6	20	5	15	1.5	7
D9	Group1 ~ 5	16	2 ~ 3	7.5	5	2.5	1.5	7
D10	Group1 ~ 5	16	2 ~ 3	20	5	15	1.5	7
D11	Group1 ~ 5	16	2 ~ 3	30	5	25	1.5	7

### 2.3 Sample collection and analysis

#### 2.3.1 Water sample analysis

Water samples were collected daily from the outlet of the EFI devices, and physical water quality parameters were measured *in situ*. DO was measured using a dissolved oxygen meter (JPB-607a, Leici, China), pH was measured with a pH meter (PHSJ-3F, Leici, China), and EC was determined using a conductivity meter (DDS-11A, Leici, China). The collected samples were transported to the laboratory for further analysis using a UV spectrophotometer (UV-2600, Daojin, Japan). Prior to analysis, the water samples were filtered through a 0.45 μm cellulose acetate membrane. COD, TN, ${\mathrm{NH}}_{4}^{+}-N,$ ${\mathrm{NO}}_{3}^{-}-N$ and ${\mathrm{NO}}_{2}^{-}-N$ were measured following standard methods,with three independent replicates (*n* = 3) for each sample. Standard deviation (SD) was used to represent the data dispersion.

#### 2.3.2 Plant analysis

The leaves of plants collected before and after the operation of the device were cleaned, weighed, and ground to analyze photosynthetic pigments, including chlorophyll a (*Chl-a*), chlorophyll b (*Chl-b*), and carotenoids (*Car*), as well as catalase (CAT). Photosynthetic pigments were extracted using the acetone method, and their concentrations were measured with a UV spectrophotometer. Catalase (CAT) was assayed using the consumption of hydrogen peroxide to determine the activity of the enzyme, and the absorption of UV–visible light by hydrogen peroxide was used to detect changes in hydrogen peroxide content. A detailed procedure for detecting the physiological properties of the plants is provided in the Appendix S2, S3.

#### 2.3.3 Microbial community analyses

To investigate changes in microbial community composition and diversity during the treatment of actual nitrogenous wastewater using immobilized microorganisms combined with the EFI, microbial samples were collected from various sources. These included water samples from the worst (EFIa1) and best runs (EFIa2), around the blades of *Vallisneria natans* (a3), the roots of the plants (a4), and the Original water (a5). The sample counts are presented in Table 3. Effluent water samples from the EFI device were filtered using 0.22 μm aqueous filter membranes, which were subsequently stored at −50 °C for freezing. The membranes were then analyzed for microbial community composition through 16S rRNA high-throughput sequencing. Shanghai Meiji Biomedical Technology Co., Ltd. conducted this portion of the experiment, which included sample pretreatment, DNA extraction, PCR amplification, preparation of standard sample curves, and data analysis.

**Table 3:** Microbiological samples and sampling conditions.

Sample	Sampling conditions
EFIa1	DO = 2 ~ 3 mg/L, HRT = 7d, C/N = 5
EFIa2	DO = 2 ~ 3 mg/L, HRT = 7d, C/N = 16
a3	Surrounding submerged plants
a4	Plant roots
a5	Original water

## 3 Results and discussion

### 3.1 Impact of C/N on operational effectiveness

**Figure 2:**
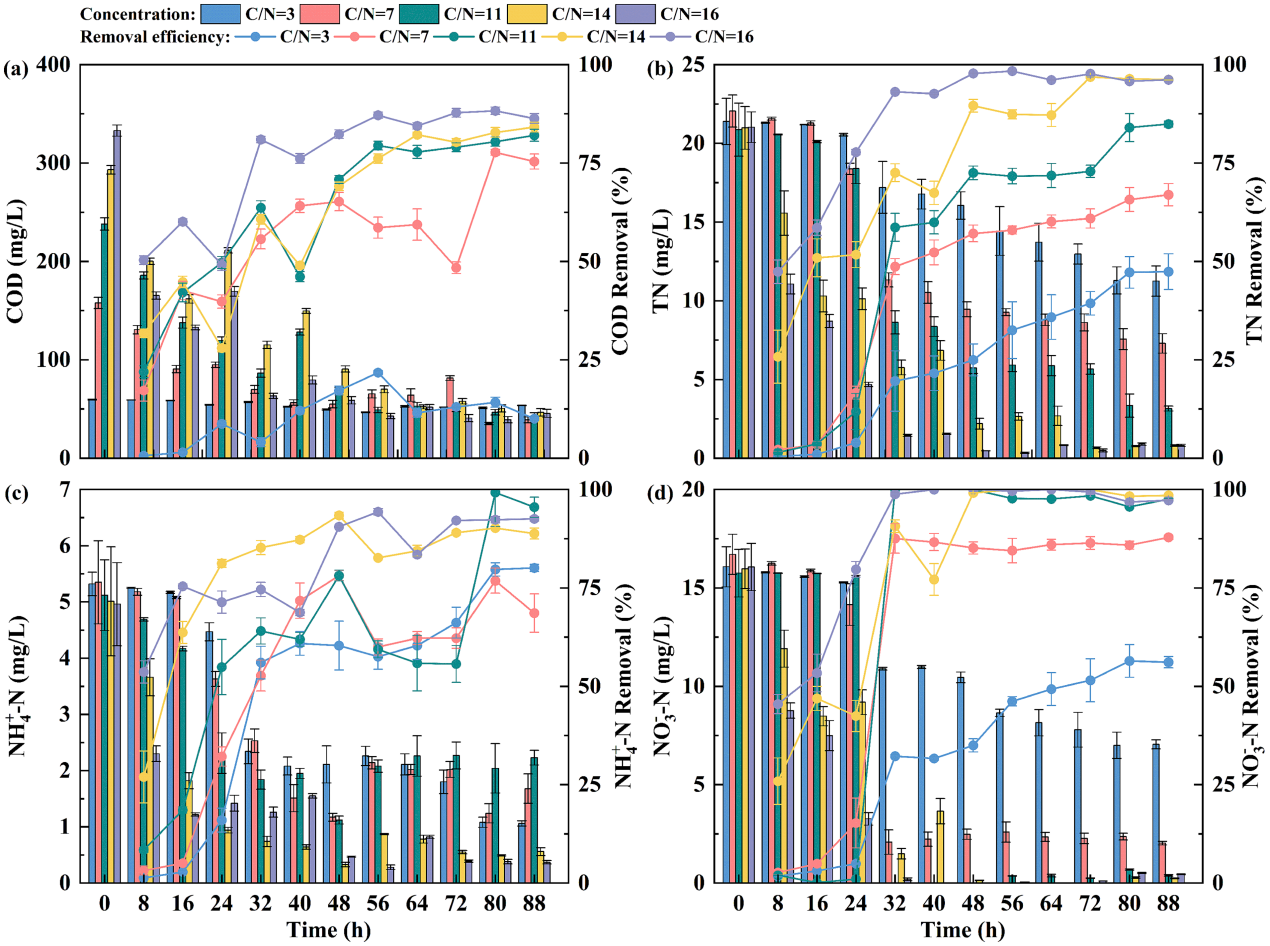
**(a)** COD, **(b)** TN, **(c)** ${\mathrm{NH}}_{4}^{+}-N$ and **(d)** ${\mathrm{NO}}_{3}^{-}-N$ removal by EFI under different C/N conditions.

From Figure 2a, it is evident that a higher C/N ratio enhances COD removal efficiency. The COD removal efficiency peaked at 88.27% when the C/N ratio was 16, while dropping to a minimum of 21.73% at a C/N ratio of 3. This demonstrates that the availability of organic carbon sources directly impacts the denitrification efficiency of denitrifying bacteria. When the carbon source is insufficient, denitrifying bacteria resort to utilizing their own protoplasm for endogenous denitrification. This process significantly reduces both the activity and population of denitrifying bacteria, thereby impairing nitrogen removal efficiency (Zhang et al., 2024a).

The nitrogen removal performance of the EFI system for nitrogenous wastewater is shown in Figure 2. As depicted in Figure 2b, the TN removal rate increased with the rise in the C/N ratio. The final TN removal rates were 47.41 ± 4.53%, 66.94 ± 2.81%, 84.91 ± 0.77%, 96.19 ± 0.19%, and 96.15 ± 0.33%, respectively. At C/N ratios of 14 and 16, the system stabilized after 48 h of operation. The removal of ${\mathrm{NH}}_{4}^{+}-N$ also showed excellent performance, achieving removal rates of 93.41 ± 0.79% and 90.52 ± 0.20%, respectively, as shown in Figure 2c. However, at C/N ratios of 3 and 7, the removal rate of ${\mathrm{NH}}_{4}^{+}-N$ initially increased and then gradually stabilized, with final removal rates of 80.07 ± 0.88% and 68.59 ± 4.90%, respectively. This lower removal efficiency is attributed to the insufficient C/N ratio. The limited availability of organic matter restricts the activity of denitrifying bacteria, inhibiting the reduction of ${\mathrm{NO}}_{2}^{-}-N$ to N_2_. Simultaneously, nitrite accumulation suppresses the activity of ammonia-oxidizing bacteria (AOB), which are responsible for oxidizing ${\mathrm{NH}}_{4}^{+}-N$ to NO_2_^−^-N, thereby hindering the overall nitrification–denitrification cycle and resulting in compromised NH_4_^+^-N removal (Dai et al., 2025). As shown in Figure 2d, during the first 32 h, the removal rate of ${\mathrm{NO}}_{3}^{-}-N$ increased with the rise in the C/N ratio and eventually stabilized. The final removal rates of ${\mathrm{NO}}_{3}^{-}-N$ were 56.12 ± 1.43%, 87.84 ± 0.54%, 97.52 ± 0.25%, 98.49 ± 0.25%, and 97.26 ± 0.13%, respectively. The immobilized denitrifying bacteria demonstrated superior nitrate removal performance under sufficient carbon source conditions (C/N ≥ 11), achieving a final removal rate of over 97%. However, when the C/N ratio was insufficient, the number and activity of microorganisms in the system were negatively affected, leading to a decrease in nitrogen removal efficiency. This observation aligns with the findings of other researchers. Sun et al. (2023) used a composite membrane aerated biofilm reactor (MABR) to treat salt-containing wastewater at different C/N ratios. Their results showed that when the C/N ratios were 6, 4.5, and 3, the TN removal efficiencies of the MABR system were 73.21, 69.51, and 67.04%, respectively. These findings indicated that TN removal efficiency decreased as the C/N ratio declined. Similarly, Allen et al. (2023) explored the effect of varying C/N ratios on the removal of total dissolved nitrogen (TDN) in laboratory experiments. Their results showed that TDN was almost completely removed at the highest C/N ratio, with higher C/N levels enhancing denitrification and improving TN removal efficiency.

### 3.2 Impact of DO on operational efficiency

All EFI devices achieved COD removal rates above 77.13% following 6 days of operation, with effluent COD concentrations between 63.71 and 71.30 mg/L (Figure 2a). Notably, the 2 ~ 3 mg/L DO group exhibited significantly superior and more stable COD removal efficiency relative to other DO conditions.

**Figure 3:**
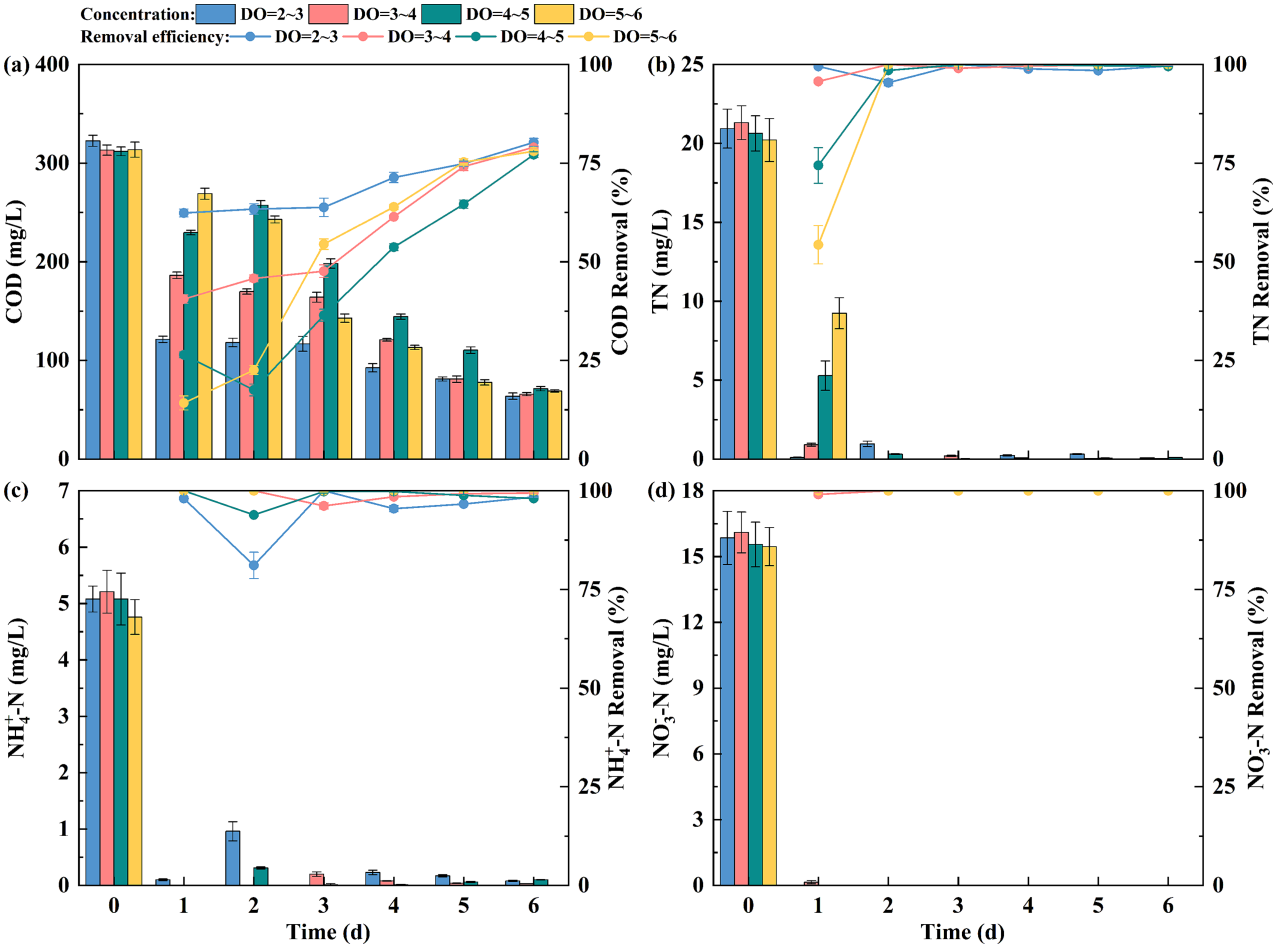
**(a)** COD, **(b)** TN, **(c)** ${\mathrm{NH}}_{4}^{+}-N,$ and **(d)** ${\mathrm{NO}}_{3}^{-}-N$ removal by EFI under different DO conditions.

The removal efficiency of EFI on nitrogenous pollutants in water is shown in Figure 3. Different DO concentrations in EFI resulted in improved removal of nitrogenous pollutants, with removal rates for TN, ${\mathrm{NH}}_{4}^{+}-N,$ and ${\mathrm{NO}}_{3}^{-}-N$ exceeding 96% by the third day of operation for each system. The TN removal rate increased progressively with higher DO concentrations (Figure 3b), and this trend was primarily driven by aerobic denitrification of *Alcaligenes faecalis.* During the first 3 days of system operation, a slight accumulation of TN occurred due to the buildup of ${\mathrm{NO}}_{2}^{-}-N$ , which led to an increase in TN concentration. However, as the system continued to operate, TN was completely removed. The immobilized denitrifying bacteria combined with the EFI system exhibited enhanced removal of nitrogenous pollutants in the actual water body. Furthermore, higher DO concentrations resulted in a faster nitrogen removal rate. As the DO concentration increased, the removal rate of ${\mathrm{NH}}_{4}^{+}-N$ also improved, reaching nearly 100% on the first day of operation when DO was between 5 and 6 mg/L (Figure 3c). When DO ranged from 2 to 5 mg/L, the removal rate of ${\mathrm{NH}}_{4}^{+}-N$ decreased initially. However, by the third day of operation, the removal rate stabilized at above 96.16%. As shown in Figure 3d, the concentration of DO has little effect on the removal of ${\mathrm{NO}}_{3}^{-}-N$ , and the removal rate remains constant throughout the operation of the system. This indicates that the immobilized denitrifying bacteria exhibit a stable removal effect on ${\mathrm{NO}}_{3}^{-}-N$ . Considering both the operational cost and denitrification performance, the DO concentration has minimal influence on the removal of nitrogenous pollutants in the actual water body. However, when DO is in the range of 2 ~ 3 mg/L, the COD removal efficiency is higher than that in other groups. Therefore, subsequent experiments were conducted using a DO concentration of 2 ~ 3 mg/L.

### 3.3 Impact of influent concentration on operational efficiency

**Figure 4:**
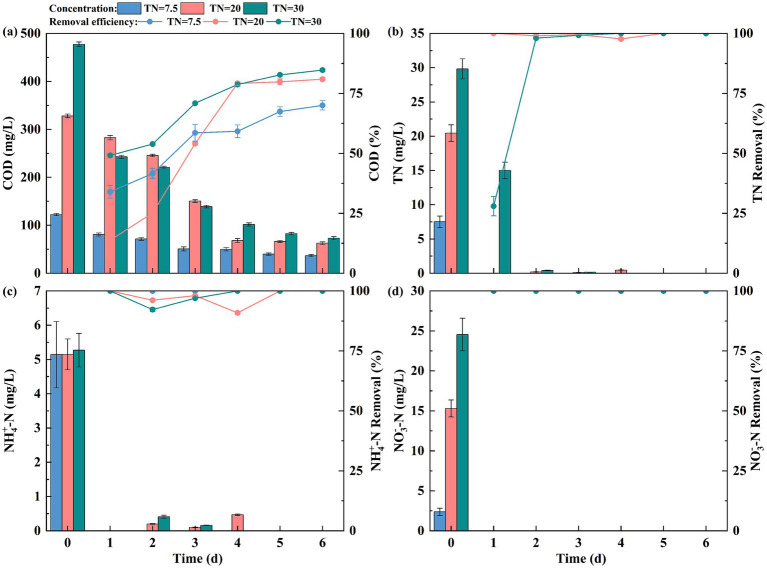
**(a)** COD, **(b)** TN, **(c)** ${\mathrm{NH}}_{4}^{+}-N,$ and **(d)** ${\mathrm{NO}}_{3}^{-}-N$ removal by EFI under different TN conditions.

As illustrated in Figure 4a, COD removal rates of the EFI system stabilized after 4 days of operation with increasing influent nitrogen concentrations, with final efficiencies of 70.00 ± 1.93%, 80.90 ± 0.70%, and 84.69 ± 0.66% for the three tested TN levels. When the TN concentration in the influent water was 30 mg/L, the COD removal was more effective compared to other influent concentrations, indicating that the device can efficiently remove organic matter from contaminated water. However, when the TN concentration was 7.5 mg/L, the COD removal was the least effective. This phenomenon is a direct consequence of our experimental design, in which a fixed optimal C/N ratio of 16 was maintained across all groups; at the low influent TN concentration of 7.5 mg/L, this design resulted in a low organic matter concentration in the system, which could reduce the number and activity of microorganisms, thereby influencing the COD removal performance (Zhang Q. et al., 2024).

The removal efficiency of the EFI on actual nitrogenous wastewater is shown in Figure 4. As illustrated in Figure 4b, Complete and stable nitrogen removal was achieved on the first day for the 7.5 mg/L influent TN group. For 20 mg/L and 30 mg/L influent TN, nitrification-induced NO₂^−^-N accumulation occurred initially, but sufficient organic matter and DO supported efficient denitrification of both ${\mathrm{NO}}_{2}^{-}-N$ and ${\mathrm{NO}}_{3}^{-}-N$ , resulting in 100% final TN removal. As shown in Figure 4c, as the concentration of nitrogen pollutants in the influent water increased, the ${\mathrm{NH}}_{4}^{+}-N$ concentration decreased rapidly, with complete removal occurring after 5 days. When the influent TN was 7.5 mg/L, ${\mathrm{NH}}_{4}^{+}-N$ was removed on the first day of system operation. For influent TN concentrations of 20 mg/L and 30 mg/L, a small amount of ${\mathrm{NH}}_{4}^{+}-N$ remained on day 4, but it was completely removed as the system continued to operate. This indicates that effective removal of ${\mathrm{NH}}_{4}^{+}-N$ can be achieved at varying influent nitrogen concentrations. When the influent nitrogen concentration was between 7.5 and 30 mg/L, ${\mathrm{NO}}_{3}^{-}-N$ was completely removed on the first day of system operation, and the removal rate remained consistent throughout the operation (Figure 4d). As a result, the device is highly effective in treating both organic matter and nitrogen in eutrophic actual water bodies.

### 3.4 Analysis of plant physiological and biochemical indicators

**Figure 5:**
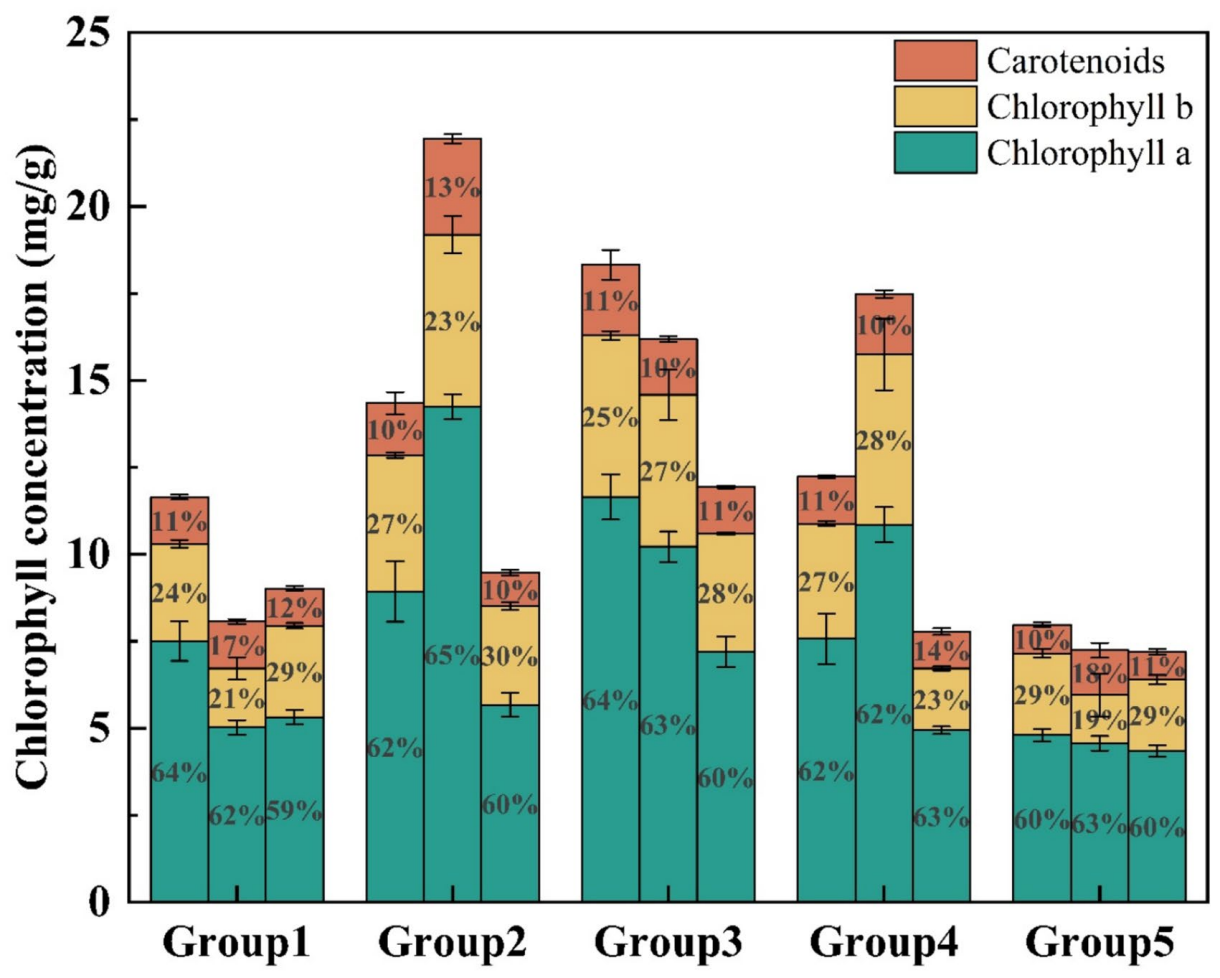
Chlorophyll content across different plant groups.

As shown in Figure 5, a comparison of chlorophyll content in the EFI system across different plant groups revealed that Chl-a accounted for the largest proportion (60 to 65%), followed by Chl-b (19 to 29%) and Car (10 to 17%). *Canna indica* L. exhibited high chlorophyll content in the first 3 groups. It grew particularly well in the EFI system, with its strong root system allowing it to attach more microorganisms and absorb nutrients effectively (Singh et al., 2025). In groups 2 and 3, plant pairings showed higher overall chlorophyll content, with *Canna indica* L. in group 2 showing a total chlorophyll content of 12.85 mg/g, and *Canna indica* L. in group 3 reaching 16.29 mg/g. Additionally, *Thalia dealbata Fraser* in group 2 had significantly higher chlorophyll content compared to the other plants, at 19.19 mg/g. *Myriophyllum verticillatum* L. also showed higher chlorophyll content, which can be attributed to the fact that, over time, the roots of *Myriophyllum verticillatum* L. in the floating island system reached the bottom of the device and made contact with the sediment. This interaction likely promoted the reproduction of foxtail algae, resulting in significant growth. In contrast, the chlorophyll content of *Vallisneria natans* did not vary significantly across all five plant combinations. This is likely due to the fact that *Vallisneria natans* grows at the bottom of the device, where changes in external environmental factors, such as a decrease in light, might alter the light compensation point, thereby regulating the chlorophyll content of the plant (Chou et al., 2022).

**Figure 6:**
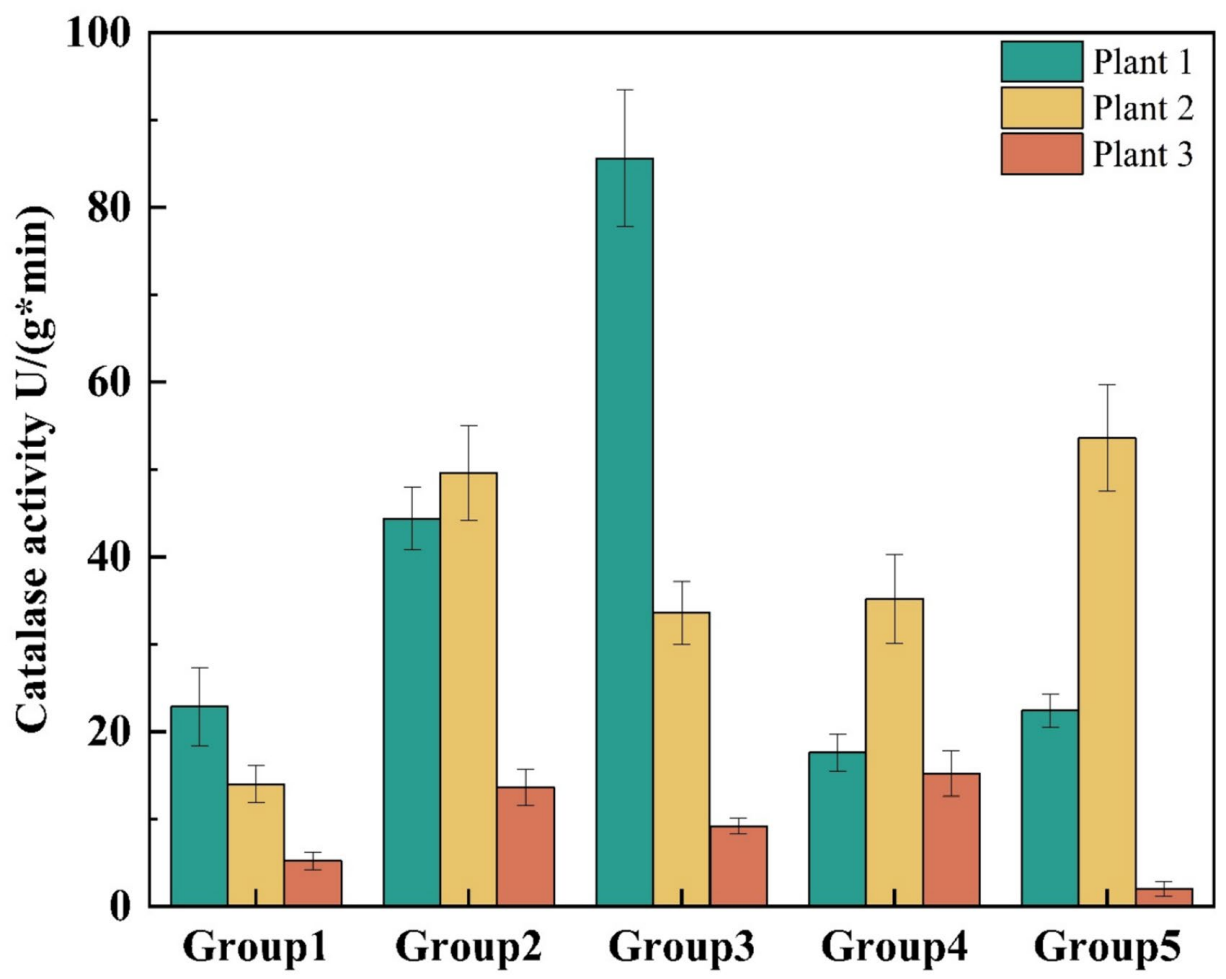
Changes in CAT activity in different plant groups.

CAT plays an important role in plant growth and development, serving as an important enzymatic antioxidant that catalyzes the decomposition of H_2_O_2_ into O_2_ and H_2_O (Mhamdi et al., 2012; Mittler et al., 2022). The CAT content in plants can reflect their resistance and resilience to environmental stress (Yu et al., 2024b). To visualize changes in CAT activity across different plant groups in each device, the data from 4 min were compared. As shown in Figure 6, the CAT enzyme activity in group 2 and group 3 was higher than in the other groups. This can be attributed to the presence of *Canna indica* L. and *Thalia dealbata Fraser*, both of which have well-developed root systems and taller structures, giving them a greater ability to resist stress. Additionally, *Myriophyllum verticillatum* L. in group 3 and group 4 showed better stress resistance, as the plant’s roots reached the bottom of the device, providing it with a better survival space.

### 3.5 Analysis of the microbial community structure

Table 4 shows the microbial richness and diversity indices for each sample. The Coverage index for all sample was greater than 0.99, indicating that the coverage rate of the water samples exceeded 99%, ensuring a high level of coverage and meeting the requirements for subsequent analysis (Qian-Qian et al., 2021). The Chao1 index, in order of magnitude, was EFIa2>EFIa1>a3>a4>a5, indicating that microbial abundance was higher under optimal operating conditions compared to suboptimal conditions. Microbial abundance significantly increased when both carbon sources and DO were sufficient. Additionally, the microbial abundance in the water body was greater than that around the *Vallisneria natans* leaves and plant roots, with the microbial abundance in the raw water being significantly lower than that in the device system. Regarding microbial community diversity, the Shannon index, in order of magnitude, was a5 > a3 > EFIa1 > EFIa2 > a4, while the Simpson index, in order of magnitude, was EFIa2 > a4 > EFIa1 > a3 > a5. The highest microbial diversity was observed in a5, and diversity significantly decreased after the addition of immobilized microorganisms. The introduction of immobilized microorganisms led to the aggregation and rapid growth of dominant bacteria in the system, thereby reducing microbial diversity in the water body of the device. Additionally, the abundance of microorganisms around the leaves of *Vallisneria natans* was higher than around the plant roots, signifying that submerged plants enhanced the attachment of microorganisms (Yang et al., 2022).

**Table 4:** Microbial richness and diversity indices.

Sample	Ace	Chao1	Coverage	Shannon	Simpson	Sobs
EFIa1	279.8042	279.5833	0.997208	2.62874	0.186072	264
EFIa2	354.5363	347.459	0.994334	2.487553	0.208503	309
a3	271.0991	270.3226	0.997783	3.090018	0.156928	259
a4	273.5578	255.4	0.995073	2.231872	0.205197	220
a5	219.0643	216.2188	0.998193	3.394237	0.063231	209

**Figure 7:**
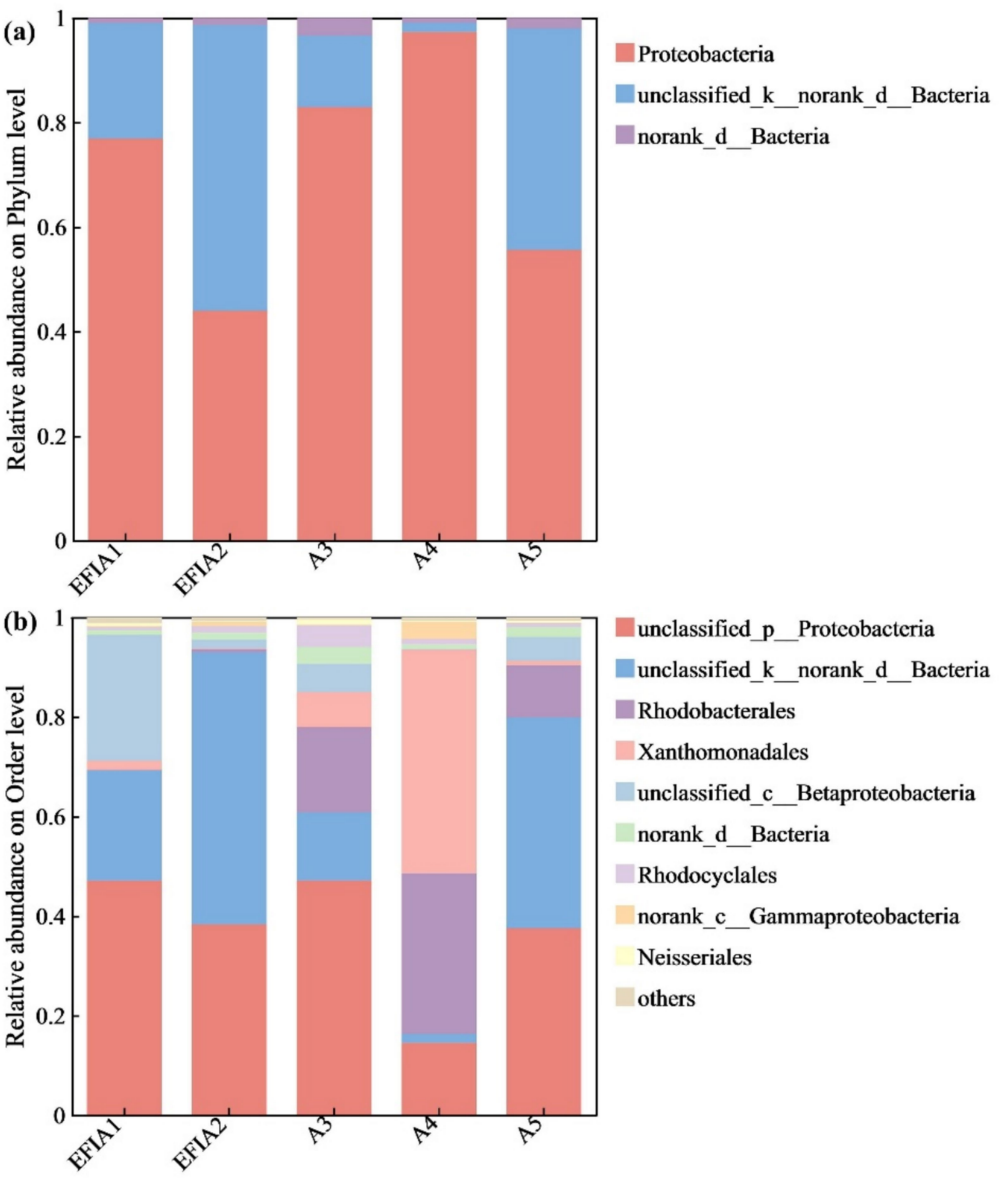
Community abundance percentages at the microbial **(a)** phylum and **(b)** order levels.

As shown in Figure 7, the microbial communities of the samples differed at the phylum level, but the overall structure was similar. The most abundant phyla across all five samples were Proteobacteria and unclassified_k_norank_d_Bacteria, while the contribution of norank_d_Bacteria was comparatively lower. The study indicated that Proteobacteria played a significant role in the improvement of nitrogenous wastewater (Su et al., 2020). The highest proportion of Proteobacteria (82.92 and 97.31%) was observed around the leaves and roots of the plants, while in the water body (EFIa1, EFIa2, a5), the percentage ranged from 44.00 to 77.91%. The content of Proteobacteria decreased in the device under the condition of a C/N ratio of 16, while the unclassified phylum of denitrifying bacteria, unclassified_k_norank_d_Bacteria, increased by 12.30%. This indicates that the unclassified denitrifying bacteria proliferated under optimal conditions, enhancing the removal efficiency of nitrogen and phosphorus pollutants in the system.

At the order level, the microbial diversity around the leaves and plant roots of *Vallisneria natans* differed significantly from that of the other samples, with a notable presence of Xanthomonadales, an order of *γ*-amoebae, which accounted for 7.05 and 44.90%, respectively. The percentage of unclassified_k_norank_d_Bacteria around the plant roots was smaller compared to the other samples, but it increased significantly when the system was under optimal operating conditions. Previous studies have shown that this microorganism has a strong ability to remove nitrogen. For instance, Deng et al. (2023) found that unclassified_k_norank_d_Bacteria and unclassified_p_Proteobacteria regulate ${\mathrm{NO}}_{3}^{-}-N$ and ASN (aminosugar-N), respectively, with both microorganisms playing critical roles in mediating NIR activity, while nosZ/nirK (N_2_O reductase/nitrate reductase) enzymes are key in this process.

## 4 Conclusion

In this study, we investigated the effects of the C/N ratio, DO, and influent nitrogen concentration on the treatment of actual nitrogenous wastewater using an EFI combined with immobilized denitrifying bacteria *Alcaligenes faecalis*. We analyzed water quality indices, chlorophyll content, and CAT activity in the leaves of five different plant groups within the system and examined the microbial community structure under varying conditions. The results identified the optimal operating conditions for the integrated system as a C/N ratio of 16 and a DO concentration of 2 ~ 3 mg/L. Under such optimized conditions, the system exhibited COD removal efficiency ranging from 70.00 to 84.69%, while attaining 100% TN removal across the full range of tested influent TN concentrations (7.5–30 mg/L). Therefore, this integrated device demonstrated excellent treatment efficiency for both organic matter and nitrogen in eutrophic actual water bodies. The overall chlorophyll content in plants from group 2 (*Canna indica L.* + *Thalia dealbata Fraser* + *Vallisneria natans*) and group 3 (*Canna indica L.* + *Myriophyllum verticillatum L.* + *Vallisneria natans*) was higher, along with increased CAT activity compared to other groups. This indicates that these two plant combinations exhibited greater resilience to adverse conditions and better adaptability to harsh environments. The highest microbial abundance was observed in the device’s water body under optimal operating conditions (DO = 2 ~ 3 mg/L, HRT = 7d, C/N = 16). At the phylum level, the predominant groups across all five samples were unclassified_k_norank_d_Bacteria and Proteobacteria. At the order level, unclassified_p_Proteobacteria and unclassified_k_norank_d_Bacteria were the most abundant. The proportion of unclassified_k_norank_d_Bacteria increased significantly after the device operated under optimal conditions, indicating that this microorganism was the dominant species and contributed significantly to nitrogen removal. In summary, the results of this study contribute to advancing innovation and development in wastewater treatment technologies. They provide valuable insights and novel approaches for improving the performance of EFI systems in water treatment applications.

## Data Availability

The datasets presented in this study can be found in online repositories. The names of the repository/repositories and accession number(s) can be found in the article/Supplementary material.
