# Supplementary material for: Performance of immobilized denitrifying bacteria-enhanced ecological floating island for treating actual nitrogenous wastewater
Source: Front Microbiol. 2026 Mar 27;17:1785618. doi: 10.3389/fmicb.2026.1785618 (PMC13066192; doi:10.3389/fmicb.2026.1785618)
Supplement: Supplementary file 1 [file Data_Sheet_1.DOCX]

**Supplementary materials**

Number of pages: 6

Number of texts: 3

Contents:

Text:

- Text S1
- Text S2
- Text S3

**Text S1. Preparation process for the immobilized *Alcaligenes faecalis* spheres**

**Preparation of bacterial suspension:** Heterotrophic nitrification-aerobic denitrification bacteria *A. faecalis* (lyophilized strain) was purchased from the China Center of Industrial Culture Collection (strain number: CICC23439). The culture solution of *A. faecalis* consists of 0.2 g KNO_3_, 0.5 g sodium citrate, 0.5 g peptone, 0.3 g beef extract, 0.5 g NaCl, and 100 mL sterilized distilled water, with a pH ranging from 7.0-7.2. The lyophilized strain was kept at -80°C until use, then a pipette was used to transfer 5 mL of sterilized nutrient broth into a glass tube containing the A. faecalis lyophilized strain, which was then shaken and sealed with sterile sealing film, and place in a 28°C, 150 r/min constant temperature shaker for 72 h. After two repeated cultivations following this method, the microorganisms were inoculated into the denitrification culture solution at a 1% (volume ratio) inoculation rate on a super-clean bench for cultivation, resulting in an enriched culture solution of *A. faecalis*. The enrichment culture was centrifuged at 5000 rpm and 5°C for 10 min and the OD600 was measured. The bacterial suspension was then diluted to an OD600 of ~1 and stored in a 4°C refrigerator for subsequent experiments.

**The preparation of immobilized microorganisms was as follows:** (1) 4 g polyvinyl alcohol and 2.5 g sodium alginate were added to 100 mL ultrapure water and slowly heated to 85°C under continuous stirring until the mixture was evenly mixed. (2) The mixed solution was placed in a high-pressure sterilization pot for sterilization then on a super-clean bench and subjected to ultraviolet disinfection. The mixed solution was cooled at room temperature and a glass rod was used to eliminate the bubbles generated during the preparation process. (3) After the solution cooled, the corresponding amount of *A. faecalis* bacterial suspension (Bacterial suspension/Mixed solution=2/1) was added and stirred to a homogeneous mixture, which was then squeezed to a 2% mass fraction of CaCl_2_ solution through a syringe. The crosslinking reaction ran for 2 h, forming small spheres with a diameter of ~5 mm. After washing the immobilized beads with sterile distilled water, they were left to stand at 4℃ for 24 h.

**Text S2. Photosynthetic pigment determination**

Photosynthetic pigment measurements were performed according to a previously reported method. The fresh leaf was ground to powder in an ice bath mortar, extracted using 3 mL of 96% (v/v) ethanol, rinsed and resuspended in 10 mL. The homogenate was transferred to a centrifuge tube and stored in the dark at 4°C for 24 h before being centrifuged at 3500 rpm for 10 min. The precipitate was washed and collected with the supernatant, then diluted to form a final volume of 20 mL. The absorbance was measured at 470, 649 and 665 nm using 96% (v/v) ethanol as the reference solution. The values for each element were calculated as follows:

C_a_ = 13.95×A_665_ - 6.88×A_649_

C_b_ = 24.96×A_649_ - 7.32×A_665_

C_c_ = (1000×A_470_ - 2.05×C_a_ -114.8×C_b_)/245 (mg/L)

Where, C_a_ represents chlorophyll a; C_b_ represents chlorophyll b; and C_c_ represents carotenoid.

**Text S3. Determination of CAT**

**Enzyme solution preparation:** 0.1 g of fresh leaves were washed in a pre-cooled mortar with 5 mL 50 mmol/L pre-cooled phosphate buffer solution (pH 7.8) and then ground, homogenized and fixed. For further analysis, the enzyme solution was transferred to a centrifuge tube and centrifuged at 4°C and 6000 rpm for 15 min.

**CAT:** The measurement of CAT activity was based on the rate of H_2_O_2_ decomposition, with absorbance readings (240 nm) taken at 3 min intervals. 100 µL of the enzyme reaction solution was added to 2 mL of 50 mM phosphate buffer solution (pH 7.0) and 1 mL of 30 mM H_2_O_2_. The absorbance value was recorded every 10 s (measured for 40 s).

CAT = [ΔA_240_×V_t_] / (W×V_s_×0.01×t) (U/g min FW)
